# Evaluation of Store Environment Changes of an In-Store Intervention to Promote Fruits and Vegetables in Latino/Hispanic-Focused Food Stores

**DOI:** 10.3390/ijerph17010065

**Published:** 2019-12-20

**Authors:** Jennifer Sanchez-Flack, Barbara Baquero, Shih-Fan Lin, George Belch, Julie L. Pickrel, Cheryl A. M. Anderson, Elva Arredondo, Maria Elena Martinez, Joni Mayer, Ming Ji, John P. Elder, Guadalupe X. Ayala

**Affiliations:** 1 Institute for Health Research and Policy, University of Illinois at Chicago 1747 West Roosevelt Road, WROB 478, Chicago, IL 60608, USA; 2 Health Services, School of Public Health, University of Washington, Box 35480, Seattle, WA 98195, USA; bbaquero@uw.edu; 3 Institute for Behavioral and Community Health, San Diego State University Research Foundation, 9245 Sky Park Court, San Diego, CA 92123, USA; slin@sdsu.edu (S.-F.L.); jpickrel@sdsu.edu (J.L.P.); 4 Marketing Department, Fowler College of Business, San Diego State University, 5500 Campanile Drive, San Diego, CA 92183, USA; gbelch@sdsu.edu; 5 Family Medicine and Public Health, University of California, San Diego, 9500 Gilman Drive, La Jolla, CA 92093, USA; c1anderson@ucsd.edu (C.A.M.A.); e8martinez@ucsd.edu (M.E.M.); 6 Division of Health Promotion and Behavioral Science, School of Public Health, San Diego State University Institute for Behavioral and Community Health, San Diego State University Research Foundation, 9245 Sky Park Court, San Diego, CA 92123, USA; earredon@sdsu.edu (E.A.); jmayer@sdsu.edu (J.M.); jelder@sdsu.edu (J.P.E.); ayala@sdsu.edu (G.X.A.); 7 College of Nursing, University of South Florida, 12901 Bruce B Downs Blvd MDC Box 32, Tampa, FL 33612, USA; mji@health.usf.edu

**Keywords:** in-store intervention, Latinos/Hispanics, consumer food environment, retail food environment, healthy food promotion

## Abstract

Implementing interventions that manipulate food store environments are one potential strategy for improving dietary behaviors. The present study evaluated intervention effects, from the *El Valor de Nuestra Salud* (The Value of Our Health) study, on in-store environmental changes within Latino/Hispanic-focused food stores (*tiendas*). Sixteen *tiendas* were randomly assigned to either: a six-month structural and social food store intervention or a wait-list control condition. Store-level environmental measures of product availability, placement, and promotion were assessed monthly from baseline through six-months post-baseline using store audits. Linear mixed effects models tested for condition-by-time interactions in store-level environmental measures. Results demonstrated that the intervention was successful at increasing the total number of fruit and vegetable (FV) promotions (*p* < 0.001) and the number of FV promotions outside the produce department (*p* < 0.001) among *tiendas* in the intervention versus control condition. No changes in product availability or placement were observed. Results suggests changing the marketing mix element of promotions within small stores is measurable and feasible in an in-store intervention. Difficulties in capturing changes in product availability and placement may be due to intervention implementation methods chosen by *tiendas*. It is important to build upon the lessons learned from these types of interventions to disseminate evidence-based in-store interventions.

## 1. Introduction

The importance of the in-store food environment on food purchasing is supported by previous cross-sectional and intervention research. For example, shelf space [1,2,3], number of store displays [2,4,5], and promotions [6,7] influence customers’ purchasing of foods and beverages. Previous interventions that used information-only strategies via print promotions (e.g., shelf labels) observed an increase in sales of targeted foods, including fruits and vegetables (FVs); however, one promotion-related study found no intervention effects on sales of FVs [8]. Furthermore, interventions that increased the availability of FVs did not always find increased purchases of these foods [9]. For example, one systematic review reported that several interventions targeting the availability of FVs (e.g., increasing the number of FVs stocked) observed increases in the overall sales or individual-level purchases of healthy foods such as FVs, however, two interventions using FV availability approaches observed no improvement in the overall purchasing of healthy foods such as FVs [9]. In a separate systematic review, mixed results were reported for interventions targeting the placement or location of FVs throughout the store (e.g., FVs placed in the front versus the back of the store) and the impact on purchases [10]. One intervention placed FV displays near the front of the store to increase their visibility and reported increases in FV purchases among customers; however, other similar interventions reported no improvement in FV purchases [10,11]. A different systematic review concluded that multipronged strategies to increase both the supply (e.g., availability of healthy foods) and demand (e.g., strategies to encourage purchasing at the point-of-purchase [POP]) of FVs were more likely to increase purchases of FV among customers than in-store interventions using single strategies to increase either supply or demand [12]. In fact, intervention trials utilizing multipronged strategies observed increases of 25–50% in produce sales based on sales data [12]. This is significant given previous research indicating that food purchases may be reflective of one’s overall dietary intake [13]. Therefore, in-store interventions to promote purchasing and consumption of FVs is a viable community-level intervention to improve diet. However, given the mixed evidence, it is important to identify whether in-store intervention strategies are effectively implemented to further understand the influence of such strategies on diet.

In-store interventions are often conceptualized using the Model of Community Nutrition Environments [14]. This model postulates that in-store environmental characteristics, such as the availability and promotion of healthy and unhealthy foods and beverages, may have direct or indirect influences on food purchasing [14]. Likewise, Rose et al.’s multi-dimensional conceptual model posits that in-store environmental characteristics can influence food purchasing via characteristics such as increasing shelf space for targeted products [15]. These models, in conjunction with the key strategic elements of the marketing mix (product availability, placement, promotion, and price) [16], are often used to inform in-store interventions targeting the purchase of healthy foods such as FVs. To our knowledge, limited research has examined the impact of intervention strategies on marketing mix elements related to FVs at the store environment-level. Understanding which marketing mix elements are most amendable to change is important for the dissemination and implementation of in-store interventions, and ultimately, to improve dietary behaviors [17].

Previous studies and systematic reviews on in-store interventions provide evidence for the feasibility of implementation and potential to improve the food store environment [8,9,10,12,18,19]. In studies that aimed to increase the availability of healthier foods such as FVs, low-fat milk, and low-fat baked goods in food stores, they were able to successfully increase the overall number of these products within targeted stores based on observed data [20,21,22,23]. Similarly, an intervention to promote the sales of FVs reported success in increasing measured shelf space for these foods in a large discount supermarket [24]. Additionally, numerous studies have previously reported success in increasing the number of print promotions (e.g., posters, shelf labels) for healthier foods within stores based on store audits [12,25,26,27,28]. The objective of the present study was to evaluate intervention effects on in-store environmental measures including product availability, placement, and promotion using data from *El Valor de Nuestra Salud* (The Value of Our Health; *El Valor, hereafter*).

*El Valor* was an in-store intervention that tracked changes in customers’ purchasing and intake among those serving on an evaluation cohort (Ayala et al., in preparation). The study occurred in Latino/Hispanic-focused food stores, otherwise known as *tiendas* [29]. The hypotheses for the present study were that *tiendas* in the intervention condition would have increases in:

1. product availability (i.e., increases in the number of overall and intervention-targeted fresh, canned, and frozen FVs and varieties of fresh FVs available);
2. placement (i.e., increases in the amount of shelf space dedicated to fresh FVs and number of fresh FV displays);
3. promotion of FVs (i.e., number of FV promotions overall and number of FV promotions outside the produce department [cross-product category promotions])

than *tiendas* in the control condition from baseline to six-months post-baseline (assessed via monthly store audits).

## 2. Materials and Methods

### 2.1. Design and Setting

*El Valor* was a clustered randomized controlled trial that used structural and social intervention strategies to modify the physical and social environments of *tiendas* to improve FV consumption among Latino/Hispanic customers [30]. The trial included 16-pair matched *tiendas* in San Diego County, California where approximately 33% of the population is of Latino/Hispanic origin [31]. Pair-matched *tiendas* were randomized to a six-month intervention or a wait-list control condition. Store audits were conducted every month for the duration of the *tienda’s* involvement in the study, in addition to the baseline and six-month post-baseline assessments. The trial occurred between October 2011–October 2014. Due, in part, to staffing reasons, the six-month intervention, including recruitment, occurred in three waves [30] with baseline data collection occurring between October 2011 to October 2013. Study protocols were approved by San Diego State University’s Institutional Review Board.

### 2.2. Intervention Description

**Figure 1:**
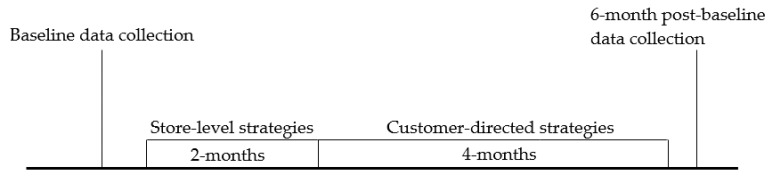
Timeline of *El Valor* intervention strategies.

*El Valor* was a six-month structural and social intervention implemented within the *tienda* environment and involved the *tiendas* owners/managers and employees. The first two months of the intervention focused on store-level strategies and the last four months were focused on customer-directed strategies. A timeline of the intervention is provided in Figure 1. Customer-directed intervention details [30] and primary outcome results are described elsewhere (Ayala et al., in preparation). Because our interest here was to determine whether the intervention was effective at modifying the targeted marketing mix elements for fresh, canned, and frozen FVs, only store-level strategies to modify the *tienda’s* environment for these FVs were examined. To increase the availability and variety of FVs, managers and employees received training on merchandising FVs throughout the *tienda*. To enhance the placement and promotion of FVs throughout the *tienda*, managers received $2000 to purchase new equipment and marketing materials. Decisions on what equipment to purchase was decided between the *tienda* manager and the research intervention coordinator. Managers were encouraged to purchase new fresh FV displays (e.g., cold food bar to promote the sale of ready-to-eat FVs) and/or hardware to improve existing FV displays (e.g., shelf extensions). The promotional strategies involved a four-month FV promotion campaign directed at the customers and included nine bi-weekly food demonstrations (latter not discussed here). The in-store promotional materials, or POP, included: (a) shelf-talkers; (b) aisle violators; (c) posters; (d) a banner; (e) a produce fact sheet; and (f) blank signs (with the *El Valor* logo) that *tienda* managers and employees could use to promote or price FVs. Some of the POP materials remained in place for the four-month period while other POP materials were rotated every two weeks to highlight the *El Valor* recipe and the FV items promoted at the food demonstration. POP materials were also placed outside of the FV department to cross-market FV with other product categories. For example, *tienda* managers were encouraged to use the POP materials in the butcher department to promote meat-vegetable pairings and to apply other cross merchandising opportunities (e.g., POP materials placed in cereal department to promote bananas). Examples of POP materials are included in Supplementary Material 1.

### 2.3. Tienda Recruitment

*Tiendas* were systematically sampled following an extensive enumeration process. The systematic enumeration was conducted using five sources: (1) county food permits, (2) the county health department directory of food retailers, (3) the Special Supplemental Nutrition Program for Women, Infants, and Children (WIC) program active vendor list, (4) the Supplement Nutrition Assistance Program (SNAP) authorized retailer list, and (5) a previous observational study conducted in the target area [32]. After the removal of duplicates, non-food stores, stores identifiable as not a *tienda* (e.g., super centers, liquor stores, etc.), excluding zip codes where 2000 Census data indicated that the proportion of Latino/Hispanic residents was less than 20%, and excluding San Diego’s South County because of competing intervention activities, 566 entries were included in the enumeration list. This was further reduced to 339 after initial telephone and internet verification for eligibility. Given time and resource constraints, during the final phases of recruitment, the study team identified four additional zip codes near the study offices that contained census tracts representing at least a 20% Latino/Hispanic population. From these areas, additional entries were added to the previously enumerated list of possible *tiendas*. After removing entries identified as non-food stores, 382 entries were available for verification.

A store screening checklist was used to determine if the store met *tienda* eligibility criteria to participate in the study. Eligibility criteria for *tiendas* were based on the following: (1) customer-base was largely Latino/Hispanic, (2) some or all employees were bilingual (English/Spanish language) or Spanish-speaking, (3) stores used bilingual (English-Spanish) and/or Spanish language in their in-store product signage, (4) stores offered products and services from Mexico and other Latin American countries, and (5) had a service butcher and a produce department. Full-service supermarkets were excluded. Of the 382 entries left on the enumeration list, 71% (n = 273) were not eligible and 1.5% (n = 6) were duplicates. An additional 26 stores were identified during ground truthing leaving 129 *tiendas* in the recruitment pool. From this recruitment pool, 84 were approached for participation and 21 refused to participate, 14 were not approached for other exclusionary reasons (e.g., owned by participating owner; proximity to another participating store) and 31 were not approached given that the recruitment goal of 16 *tiendas* was met. To minimize sources of variance across study conditions and the potential for cross-contamination, *tiendas* were pair-matched on store size, having a prepared food department, and being at least one mile away from the other *tienda* prior to data collection and randomization to study condition.

### 2.4. Tienda Data Collection Procedures

Store audits were conducted by trained research staff. Audits occurred at varying times of day, Monday through Friday. To avoid potential social desirability bias, store owners/managers were unaware of what specific days or times research staff would be conducting the audits. In addition, store owners/managers were unaware that the primary focus was on the availability and promotion of FVs during these observations. Longer store audit assessments were conducted at baseline and six-months post-baseline; abbreviated assessments were conducted in between these two time-points every month. The audit captured the availability of fresh (bulk and pre-cut), canned and frozen FVs, shelf space dedicated to fresh (bulk and pre-cut) FVs, the number of fresh (bulk and pre-cut) FVs displays available, and the promotion of FVs. To assess inter-rater reliability, 100% of baseline and 37.5% of six-month post-baseline store audits were conducted independently by two research assistants at the same time and compared.

### 2.5. Product Availability: Availability of Fresh, Canned, and Frozen FVs and Variety of Fresh FVs

The store audit assessed the availability of fresh (bulk and pre-cut), canned, and frozen FVs at all time-points. Data on the availability (dichotomous: yes [coded as ‘1’]/no [coded as ‘0′]) of fresh, canned, and frozen FVs were collected for a predetermined list of 73 fresh FVs, 16 frozen FVs, and 28 canned FVs, including frozen bags and cans of mixed FVs [33]. In the current study, availability was defined as follows: (1) the total number of unique types of fresh FVs available (e.g., papaya, banana, avocado, zucchini); (2) the total number of unique types of frozen FVs available (e.g., strawberries, broccoli); and (3) the total number of unique types of canned FVs available (e.g., peaches, beets). Availability scores were computed by summing the available fresh, frozen, and canned FVs (continuous) [34]. For example, if fresh, canned, and frozen spinach were available in a *tienda*, this was counted as three. An additional availability score was computed to capture the availability of FV items targeted in the intervention (continuous). These items were listed on recipe cards and included in the POP materials: apples, bananas, grapes, mangos, oranges, strawberries, bell peppers, broccoli, cabbage, carrots, celery, corn, garlic, green beans, green onions, leafy lettuce, mushroom, onions, peas, spinach, squash, and tomato. To compute this variable, all available fresh, canned, and frozen targeted FVs were summed. This operationalization is consistent with research demonstrating that customers are influenced by multiple exposures to a food item versus just a single exposure [35,36].

Store audits at all time-points also assessed the variety of fresh (bulk and pre-cut) FVs stocked within a *tienda* for each unique fresh FV available. For example, if apples were stocked within the *tienda*, the number of unique varieties of apples were counted (e.g., gala, honeycrisp, granny smith, fuji apples). A total variety score was computed by summing the total number of varieties of fresh FVs (all continuous) [37].

### 2.6. Product Placement: Shelf Space Dedicated to Fresh FVs and Number of Fresh FV Displays

A “Produce Display Measurement Form,” developed by the study team, was used to assess the amount of shelf space dedicated to fresh (bulk and pre-cut) FVs at all time-points. Data on the number of shelves for each display, shelf measures (continuous: length and width in feet) and level of stock within the display (categorical: 0–1/3, >1/3–2/3, >2/3–1, with 1 = fully stocked) were collected. If the display contained items that were not fresh FVs, the length and width for these areas were also recorded and later subtracted to obtain an accurate measurement of shelf space solely dedicated to fresh FVs. All measurements were rounded to the nearest inch and then recorded in feet. Displays that only stocked prepared or cooked FVs were not measured (e.g., potato salad). Total amount of shelf space dedicated to fresh FVs was computed by summing shelf measures for the entire *tienda* (continuous) [38].

Data also were collected on the number (dichotomous: present [coded as ‘1′]/not present [coded as ‘0′]) and type (categorical: one-sided display, pallet, island, promotional, other) of fresh (bulk and pre-cut) FV displays using a “Produce Display Tracking Form” developed by the study team; data were collected at all time-points. Displays that only stocked prepared or cooked FVs were not counted (e.g., potato salad). Number of FV displays present was computed by summing the total number of displays observed as present irrespective of size or location in the *tienda* (continuous) [39].

### 2.7. Product Promotion: FV Promotions

Promotions of fresh, canned, and frozen FVs were assessed using a “Fruit and Vegetable Promotions Form” at all time-points. The form captured detailed information on FV promotions inside and immediately outside the *tiendas*. Data collected assessed the location of promotions (categorical: outside of *tienda*, aisles, checkout, aisle endcaps, entrance, island, or other open space), product category of the item closest to the promotion (categorical: fresh FV, cereal and breakfast foods, snack foods, sugar-sweetened beverages, grains and dried beans, canned foods and soups, dairy, butcher, frozen foods, alcoholic beverages, prepared foods, deli, bakery, tortillas, other grocery, non-food, other), promotion type (categorical: price promotions, signage, hand-out, package add-on, theme, other), and number of promotions (continuous). Similar to previous research examining the influence of promotion exposure on dietary behaviors [40], the total number of FV promotions present was summed for each *tienda* (continuous). Given the influence of cross-product marketing on purchasing, a second variable was created to identify FV promotions found outside the produce department [41]. A variable reporting the total number of “cross-product category” FV promotions within each *tienda* was computed by summing the number of FV promotions that were outside the produce department (continuous). *Tiendas* with no promotions of FV during data collection were coded as zero for both total number of FV promotions and total number of non-FV cross-product category promotions.

### 2.8. Covariate: Tienda Size

Given the association between *tienda* size and in-store environmental characteristics such as the availability of products [42,43,44,45], the current study considered the total square footage of the *tienda*’s sales floor (continuous) as a covariate in the model building process.

### 2.9. Statistical Analyses

A summary of the measures used in the present study are available in Table 1.

All analyses were conducted using an intent-to-treat approach with *tiendas* analyzed per the condition to which they were randomized; analyses were adjusted for the potential clustering effects of *tiendas*. Data imputations were conducted for store audit data for three *tiendas* for the last monthly audit prior to the six-month post-baseline assessment. These *tiendas* were on a different data collection schedule and therefore had fewer assessments. In these cases, values from the previous data collection point were used for a conservative estimate of each marketing mix element. For this study, baseline, monthly abbreviated assessments collected during the intervention phase (months 3–5), and six-month post-baseline data were used to examine intervention effects as they were occurring in the *tienda*. Analyses were performed using SAS software, Version 9.4 of the SAS System for Windows (SAS Institute, Cary, NC, USA).

Descriptive statistics were obtained on the availability of fresh, canned, and frozen FVs, variety of available fresh FVs, availability of targeted fresh FVs, shelf space dedicated to fresh FVs, number of fresh FV displays, number of FV promotions, and *tienda* size characteristics. Differences by condition in baseline *tienda* characteristics were analyzed using an independent samples t-test for continuous variables and χ^2^ statistics for categorical variables. A *p* value of < 0.05 was used as the level of significance for all analyses. To assess inter-rater reliability of store audit data, Cohen’s Kappa statistics were computed for binary variables [46] and intraclass correlations (ICCs) were computed for continuous variables [47] for 37.5% of randomly selected store audits with reliability data.

Linear mixed effect models, using SAS PROC MIXED, were estimated to examine condition-by-time effects in the observed values for the marketing mix elements. For this analysis, each case was the data collection timepoint for each *tienda* (n = 16 *tiendas* × 6 timepoints = 96). The variability in marketing mix elements was plotted prior to running analyses to determine which time-dependent term(s) were appropriate. Based on the plots, the models included a linear time-dependent term. All models were adjusted for *tienda* size.

## 3. Results

### 3.1. Tienda Characteristics and Retention Rates

At baseline and across study conditions, the 16 *tiendas* were similar on several dimensions (see Table 2). Additionally, there were no significant differences between intervention versus control *tiendas* in size or in the marketing mix elements. In terms of *tienda* size, the mean square footage of the sales floor was 4083.4 (SD = 3694.3). As for the marketing mix elements, for product availability at baseline, the mean number of fresh (bulk and pre-cut) FVs was 48.8 (9.3), the mean number of canned and frozen FVs was 25.2 (11.1), the mean variety of fresh FVs was 73.7 (20.8), and the mean number of targeted FVs available was 26.2 (4.6) for *tiendas* in both conditions. As for placement at baseline, the mean square footage for shelf space dedicated to fresh FVs was 380.1 (230.2) and the mean number of displays was 11.7 (8.8) for *tiendas* in both conditions. For promotion at baseline, the mean number of FV promotions was 14.4 (25.9) and the mean number of non-FV cross-product category promotions was 10.9 (20.3) for *tiendas* in both conditions. At six-months post-baseline, all *tiendas* were retained.

### 3.2. Inter-rater Reliability

Kappa coefficients for baseline and six-months post-baseline store audit data ranged from 0.63–1.00 for categorical measures of product availability and placement variables, indicating substantial-to-perfect agreement [48]. ICCs ranged from 0.97–0.99 for continuous measures of product availability, placement, and promotion variables indicating excellent agreement between research staff [47].

### 3.3. Marketing Mix

Table 3 displays the raw mean values of *tienda* audit data values at baseline, during the intervention period (months 3–6), and at six-month post-baseline values for each of the marketing mix elements by study condition. Presented are results from mixed models estimating condition-by-time effects in marketing mix elements. The first part of the present study’s hypothesis was that compared to *tiendas* in the control condition, *tiendas* in the intervention condition would have increases in product availability (i.e., increases in the availability of overall and intervention-targeted fresh, canned, and frozen FVs and varieties of fresh FVs). However, non-significant condition-by-time interactions were observed for product availability. The second part of the hypothesis was that *tiendas* in the intervention condition would have increases in placement (i.e., increases in shelf space dedicated to fresh FVs and number of fresh FV displays) compared to *tiendas* in the control condition. Similar to product availability, non-significant condition-by-time interactions were observed. Lastly, the third part of the hypothesis was that *tiendas* in the intervention condition would have increases in promotion of FVs (i.e., overall number of FV promotions and number of non-FV cross-product category promotions) than *tiendas* in the control condition. Significant condition-by-time interactions were observed on FV promotions (*p* < 0.001) and non-FV cross product category promotions (*p* < 0.001). During the intervention period, the number of FV promotions increased from 10.9 at baseline to 82.5 during the intervention period, demonstrating a stark increase in FV promotions for the intervention condition. At six-months post-baseline, FV promotions for the intervention condition remained steady with a mean of 84. For the control condition, there was a non-significant increase of FV promotions; 18 at baseline, 16.8 during the intervention period, and 21 at six-months post-baseline. Similar trends were observed for non-FV cross product category promotions. For the intervention condition, the mean number of non-FV cross product category promotions increased from 6.5 at baseline to 44.5 during the intervention period. At six-months post-baseline, the mean number of non-FV cross product category promotions remained steady (42.6). For the control condition, the mean number of non-FV cross product category promotions also increased slightly with 11.1 at baseline, 8.4 during the intervention period, and 13.4 at six-months post-baseline (non-significant).

## 4. Discussion

*El Valor* was an in-store intervention aimed at increasing the purchasing and consumption of FVs among Latinos/Hispanics in San Diego County by modifying the in-store food environment. The present study’s results demonstrate that the intervention was successful in increasing the number of FV promotions and non-FV cross product category promotions in intervention compared with control *tiendas*. However, no intervention effects were observed for the overall number of available fresh, canned, and frozen FVs, varieties of fresh FVs available, availability of targeted FVs in any form, amount of shelf space dedicated to fresh FVs, and number of fresh FV displays.

These findings support research demonstrating that changing the environment of food stores by increasing the presence of marketing materials such as promotions is both feasible to achieve and measurable to observe [23,25,26,27,28,49,50,51,52]. Two previous in-store intervention studies utilizing promotion approaches, such as the use of marketing materials, achieved 75% implementation fidelity for the number of marketing materials placed within the store [51,52]. Another study reported observing changes in POP signs at mid-intervention and post-intervention [28]. However, unlike other intervention studies, *El Valor* was not successful in capturing changes in other aspects of the marketing mix elements such as the availability of fresh, canned, and frozen FVs, shelf space dedicated to fresh FVs, and fresh FV displays [11,21,23,24,26,53,54,55]. For example, two previous in-store interventions reported successful increases in the availability of fresh and/or canned FVs [53,56], while another study reported success in replacing shelf space dedicated to snack foods with shelf space dedicated to FVs [11].

In a systematic review of store owner/manager’s perspectives on in-store interventions, it was found that store owners/managers are receptive to intervention strategies that are easy to implement and fit into their current work schedule and space [57]. Increasing the presence of marketing materials is a less intrusive in-store environmental strategy for store owners/managers, which may be a reason why *El Valor* was able to improve this marketing mix element. Additionally, stores often receive incentives, such as marketing materials, from food distributors, therefore, the provision of marketing materials is a practice that store owners/managers already engage in and are likely to continue [58]. The provision of marketing material incentives by food distributors may also partially explain the slight, but non-significant, increase in FV promotions and non-FV cross product category promotions within the control condition [58]. Another possible explanation for this increase in the control condition is that small business owners, such as store owners/managers, often use innovative methods to increase profits. By agreeing to participate in *El Valor* but then being randomized to the control condition, store owners/managers may have utilized their own methods to promote FVs.

Regarding the intervention’s inability to see observable increases in product availability and placement, this may be due to the types of changes in which the *tienda* owners/managers engaged. For example, some *tienda* managers/owners chose to replace existing displays and equipment for new displays and equipment, therefore not changing the amount of shelf space dedicated to FVs and/or number of FV displays present but rather improving the quality of displays. This was partially due to space constraints to stock and/or install new products and displays, a barrier identified in other small store interventions [12,26,57,59,60]. Previous in-store interventions overcame one of these barriers by working closely with store owners/managers to commit to stocking just 1–3 additional FV varieties [26]. Additionally, *tiendas* in this study already began with a greater number of FVs available and greater shelf space dedicated to FVs compared to other small stores involved in intervention research [34,37,42,61]. Therefore, improving methods to capture alternative changes in FV availability and placement (e.g., measures that capture the quality of a replacement FV display) in stores that already stock FVs need to be developed and utilized in food environment research.

Another barrier to increasing product availability and placement for healthy foods, such as FVs, is time and effort [57]. Store owners/managers are more likely to stock unhealthy foods because often the distributors of these foods provide direct assistance with the delivery and stocking of these foods [57]. In future intervention trials, potential strategies to successfully improve the availability and placement of FVs may include using adaptive intervention approaches. Adaptive intervention approaches include a pre-determined sequence of decision rules that specify how the intensity or type of intervention strategy should change depending on data collected [62,63]. For example, if data collected during the intervention period demonstrates that stores are not improving product availability and placement, additional skill training and technical assistance could be provided to further support stores at improving these marketing mix elements. An adaptive intervention is not feasible in a randomized controlled trial with rolling random assignment, however, such an intervention design would work well with a Sequential Multiple Assignment Randomized Trial (SMART) design [64].

The present study does have a few limitations. *El Valor* targeted the marketing mix elements of product availability and placement, but the present study showed no improvement in those elements. This may be due to a mismatch between the measurement approach utilized in this study and intervention implementation, which did not solely target increased stocking of FVs but also targeted quality improvements in the presentation, or displays, of FVs. Additionally, the present study did not account for differences in the locations or types of FV displays and promotions utilized, which may have varying degrees of influence on purchasing and therefore should be studied [65,66]. Strengths of the present study include the sample size, its cluster randomized controlled design, and its use of systematic data collection via objective measurements of the in-store environment. Additionally, this study fills a research gap as it focused on *tiendas,* a food environment that has not been well represented in the literature in terms of in-store interventions.

## 5. Conclusions

Results suggest that changing the marketing mix element of promotions within small stores, similar to the ones used in this study, is measurable and feasible in an in-store intervention such as *El Valor*. Longitudinal studies are needed to further examine the direct influence of the marketing mix elements, such as promotions, on the purchasing of healthy and unhealthy foods. The type of food store in which such studies are conducted need to be considered as the ability to implement marketing mix strategies may differ in small stores versus supermarkets [8,10]. Supermarkets may not have the flexibility to change marketing mix elements due to their organizational policies. However, an alternative for future researchers interested in supermarkets food environment research is the use of simulated, or virtual, stores to test intervention strategies for eventual buy-in from supermarkets [10]. Additionally, formative research and/or adaptive intervention approaches should be conducted to understand how to influence product availability and placement within stores given previous evidence demonstrating the relationship between these dimensions and dietary behaviors [11,12,37,67]. One potential way to effectively manipulate multiple aspects of the in-store environment is by involving multiple partners [58,68]. For example, FV distributors and/or farmers could provide store owners/managers with technical assistance and training in acquiring and maintaining fresh FVs. Such a strategy can help build partnerships between important players in the food industry, build the capacity of stores to properly stock fresh FVs, and also foster sustainable changes [69]. Lastly, additional sensitive measures of the in-store environment need to be developed for small food stores that decide to implement alternative changes, such as the replacement of existing displays, in FV availability and placement.

## Figures and Tables

**Table 1:** Description of measures included in present study.

**Name**	**Description of Measure**
Product availability	Availability of fresh (bulk and pre-cut), canned, and frozen FVs - Number of unique fresh (bulk and pre-cut) FVs - Number of unique canned and frozen FVs - Total variety of fresh FVs - Number of intervention- targeted FVs (all forms)
Product placement	Amount of shelf space dedicated to fresh (bulk and pre-cut) FVs - Square footage of shelf dedicated to fresh FVs - Number of fresh FV displays
Product promotion	Promotions of fresh, canned, and frozen FVs - Number of intervention and non-intervention FV promotions (all types) - Number of FVs promotions outside of the produce department (non-FV cross-product category promotions)
*Tienda* size	Total square footage of the *tienda*’s sales floor

**Table 2:** *El Valor de Nuestra Salud* (The Value of Our Health) baseline *tienda* characteristics.

	All *tiendas* (N = 16)	Intervention (n = 8)	Control (n = 8)	*p*-Value ^1^
	Mean (SD)	Mean (SD)	Mean (SD)	
*Tienda characteristics*				
Number of cash registers	3.0 (1.4)	3.1 (1.4)	2.9 (1.5)	0.74
Number of aisles	4.6 (1.9)	4.8 (2.2)	4.4 (1.8)	0.71
Sales floor square footage	4083.4 (3694.3)	3899.7 (3187.7)	4267.0 (4359.7)	0.85
*Marketing mix elements*				
Product Availability				
Number of fresh (bulk and pre-cut) FVs available	48.8 (9.3)	50.1 (7.1)	47.4 (11.5)	0.57
Number of canned and frozen FVs available	25.2 (11.1)	22.6 (8.2)	27.8 (13.4)	0.37
Variety of fresh FVs available	73.7 (20.8)	74.1 (14.2)	73.3 (27.0)	0.94
Number of targeted FVs available (all forms)	26.2 (4.6)	26.9 (3.9)	25.5 (5.4)	0.57
Placement				
Shelf space dedicated to fresh FVs (sq.ft.)	380.1 (230.2)	390.5 (220.0)	371.4 (252.8)	0.87
Number of fresh FV displays	11.7 (8.8)	11.5 (6.6)	11.9 (11.1)	0.94
Promotion				
Number of FV promotions (all types, intervention and non-intervention)	14.4 (25.9)	10.9 (10.5)	18.0 (36.1)	0.60
Number of non-FV cross-product category promotions	10.9 (20.3)	7.4 (5.9)	14.8 (30.2)	0.64

^1^ Independent samples *t*-test for continuous variables, χ^2^ test for categorical variables.

**Table 3:** Results of mixed effects models estimating condition-by-time effects in marketing mix elements between baseline and six-months post-baseline ^1^.

	Intervention (n = 8 *tiendas* × 6 Time Points = 48 Time Points)			Control (n = 8 *tiendas* × 6 Time Points = 48 Time Points)			Condition by Time
	Baseline	Intervention Period (Months 3–6)	6-Month Post-Baseline	Baseline	Intervention Period (Months 3–6)	6-Month Post-Baseline	*p*-Value
	Mean (SD)	Mean (SD)	Mean (SD)	Mean (SD)	Mean (SD)	Mean (SD)	Mean (SD)
Product Availability							
Number of fresh FVs available	50.1 (7.1)	49.3 (10.4)	48.1 (8.6)	47.4 (11.5)	47.8 (11.5)	48.8 (12.6)	0.543
Number of canned and frozen FVs available	22.6 (8.2)	21.9 (7.0)	22.1 (5.6)	27.8 (13.4)	28.4 (14.2)	28.6 (14.2)	0.651
Variety of fresh FVs available	74.1 (14.2)	74.0 (20.7)	73.0 (17.6)	73.3 (27.0)	73.2 (25.1)	75.4 (29.1)	0.713
Number of targeted FVs available (all forms)	27.1 (3.9)	26.7 (4.6)	27.4 (4.5)	25.6 (5.4)	26.8 (5.3)	27.6 (5.3)	0.153
Placement							
Shelf space dedicated to fresh FVs (sq. ft.)	390.5 (220.0)	381.2 (229.7)	385.5 (238.6)	371.4 (252.8)	385.2 (252.3)	391.2 (261.9)	0.290
Number of fresh FV displays	11.5 (6.6)	10.3 (4.2)	10.4 (3.8)	11.9 (11.1)	12.8 (11.1)	12.6 (12.4)	0.518
Promotion							
Number of FV promotions (all types, intervention and non-intervention)	10.9 (10.5)	82.5 (21.3)	84.0 (19.1)	18.0 (36.1)	16.8 (26.2)	21.0 (31.7)	<0.001
Number of cross-product category promotions	6.5 (6.0)	44.5 (21.4)	42.6 (16.4)	11.1 (26.4)	8.4 (14.3)	13.4 (20.3)	<0.001

^1^ All models were adjusted for *tienda* size.

## Supplementary Materials

The following are available online at https://www.mdpi.com/1660-4601/17/1/65/s1, Supplementary Material 1: *El Valor de Nuestra Salud* (The Value of Our Health) point-of-purchase promotional materials. Figure S1: Shelf dangler, Figure S2: Aisle violator.

## References

[1] Curhan R. (1972). The relationship between shelf space and unit sales in supermarkets. J. Mark. Res..

[2] Sanchez-Flack J., Pickrel J.L., Belch G., Lin S.-F., Anderson C.A.M., Martinez M.E., Arredondo E.M., Ayala G.X. (2017). Examination of the Relationship between In-Store Environmental Factors and Fruit and Vegetable Purchasing among Hispanics. Int. J. Environ. Res. Public Health.

[3] Caspi C.E., Lenk K., Pelletier J.E., Barnes T.L., Harnack L., Erickson D.J., Laska M.N. (2017). Association between store food environment and customer purchases in small grocery stores, gas-marts, pharmacies and dollar stores. Int. J. Behav. Nutr. Phys. Act..

[4] Wilkinson J., Mason J., Paksoy C. (1982). Assessing the impact of short-term supermarket strategy variables. J. Mark. Res..

[5] Inman J., Winer R., Ferraro R. (2009). The interplay among category characteristics, customer characteristics, and customer activities on in-store decision making. J. Mark..

[6] Chandon P., Hutchinson J.W., Bradlow E.T., Young S.H. (2009). Does In-Store Marketing Work? Effects of the Number and Position of Shelf Facings on Brand Attention and Evaluation at the Point of Purchase. J. Mark..

[7] Cairns G., Angus K., Hastings G., Caraher M. (2013). Systematic reviews of the evidence on the nature, extent and effects of food marketing to children. A retrospective summary. Appetite.

[8] Adam A., Jensen J.D. (2016). What is the effectiveness of obesity related interventions at retail grocery stores and supermarkets—A systematic review. BMC Public Health.

[9] Langellier B.A., Garza J.R., Prelip M.L., Glik D., Brookmeyer R., Ortega A.N. (2013). Corner Store Inventories, Purchases, and Strategies for Intervention: A Review of the Literature. Calif. J. Health Promot..

[10] Hartmann-Boyce J., Bianchi F., Piernas C., Riches S.P., Frie K., Nourse R., Jebb S.A. (2018). Grocery store interventions to change food purchasing behaviors: A systematic review of randomized controlled trials. Am. J. Clin. Nutr..

[11] Bucher T., Collins C., Rollo M.E., McCaffrey T.A., De Vlieger N., Van der Bend D., Truby H., Perez-Cueto F.J.A. (2016). Nudging consumers towards healthier choices: A systematic review of positional influences on food choice. Int. J. Environ. Res. Public Health.

[12] Gittelsohn J., Rowan M., Gadhoke P., Gadjoke P. (2012). Interventions in small food stores to change the food environment, improve diet, and reduce risk of chronic disease. Prev. Chronic Dis..

[13] Appelhans B.M., French S.A., Tangney C.C., Powell L.M., Wang Y. (2017). To what extent do food purchases reflect shoppers’ diet quality and nutrient intake?. Int. J. Behav. Nutr. Phys. Act..

[14] Glanz K., Sallis J.F., Saelens B.E., Frank L.D. (2005). Healthy nutrition environments: Concepts and measures. Am. J. Health Promot..

[15] Rose D., Bodor J.N., Hutchinson P.L., Swalm C.M. (2010). The Importance of a Multi-Dimensional Approach for Studying the Links between Food Access and Consumption. J. Nutr..

[16] Kotler P., Armstrong G. (2010). Principles of Marketing.

[17] Story M., Kaphingst K.M., Robinson-O’Brien R., Glanz K. (2008). Creating healthy food and eating environments: Policy and environmental approaches. Annu. Rev. Public Health.

[18] Liberato S.C., Bailie R., Brimblecombe J. (2014). Nutrition interventions at point-of-sale to encourage healthier food purchasing: A systematic review. BMC Public Health.

[19] Escaron A.L., Meinen A.M., Nitzke S.A., Martinez-Donate A.P. (2013). Supermarket and grocery store-based interventions to promote healthful food choices and eating practices: A systematic review. Prev. Chronic Dis..

[20] Baquero B., Ayala G.X., Arredondo E.M., Campbell N.R., Slymen D.J., Gallo L., Elder J.P. (2009). Secretos de la Buena Vida: Processes of dietary change via a tailored nutrition communication intervention for Latinas. Health Educ. Res..

[21] Lawman H.G., Vander Veur S., Mallya G., McCoy T.A., Wojtanowski A., Colby L., Sanders T.A., Lent M.R., Sandoval B.A., Sherman S. (2015). Changes in quantity, spending, and nutritional characteristics of adult, adolescent and child urban corner store purchases after an environmental intervention. Prev. Med..

[22] Foster G.D., Karpyn A., Wojtanowski A.C., Davis E., Weiss S., Brensinger C., Tierney A., Guo W., Brown J., Spross C. (2014). Placement and promotion strategies to increase sales of healthier products in supermarkets in low-income, ethnically diverse neighborhoods: A randomized controlled trial. Am. J. Clin. Nutr..

[23] Gittelsohn J., Trude A.C., Poirier L., Ross A., Ruggiero C., Schwendler T., Steeves E.A., Powell L. (2017). The Impact of a Multi-Level Multi-Component Childhood Obesity Prevention Intervention on Healthy Food Availability, Sales, and Purchasing in a Low-Income Urban Area. Int. J. Environ. Res. Public Health.

[24] Toft U., Winkler L.L., Mikkelsen B.E., Bloch P., Glümer C. (2017). Discounts on fruit and vegetables combined with a space management intervention increased sales in supermarkets. Eur. J. Clin. Nutr..

[25] Gamburzew A., Darcel N., Gazan R., Dubois C., Maillot M., Tome D., Raffin S., Darmon N. (2016). In-store marketing of inexpensive foods with good nutritional quality in disadvantaged neighborhoods: Increased awareness, understanding, and purchasing. Int. J. Behav. Nutr. Phys. Act..

[26] Dannefer R., Williams D., Baronberg S., Silver L. (2012). Healthy bodegas: Increasing and promoting healthy foods at corner stores in New York City. Am. J. Public Health.

[27] Milliron B.-J., Woolf K., Appelhans B.M. (2012). A point-of-purchase intervention featuring in-person supermarket education impacts healthy food purchases. J. Nutr. Educ. Behav..

[28] Martínez-Donate A.P., Riggall A.J., Meinen A.M., Malecki K., Escaron A.L., Hall B., Menzies A., Garske G., Nieto F.J., Nitzke S. (2015). Evaluation of a pilot healthy eating intervention in restaurants and food stores of a rural community: A randomized community trial. BMC Public Health.

[29] Ayala G.X., Mueller K., Lopez-Madurga E., Campbell N.R., Elder J.P. (2005). Restaurant and food shopping selections among Latino women in Southern California. J. Am. Diet. Assoc..

[30] Ayala G.X., Baquero B., Pickrel J.L., Belch G., Rock C.L., Gittelsohn J., Sanchez-Flack J., Elder J.P. (2015). A store-based intervention to increase fruit and vegetable consumption: The El Valor de Nuestra Salud cluster randomized control trial. Contemp. Clin. Trials.

[31] United States Census Bureau Quick Facts San Diego County, California. https://www.census.gov/quickfacts/table/AGE275210/06073.

[32] Saelens B.E., Sallis J.F., Frank L.D., Couch S.C., Zhou C., Colburn T., Cain K.L., Chapman J., Glanz K. (2012). Obesogenic neighborhood environments, child and parent obesity: The neighborhood impact on kids study. Am. J. Prev. Med..

[33] Glanz K., Sallis J.F., Saelens B.E., Frank L.D. (2007). Nutrition Environment Measures Survey in Stores (NEMS-S) Development and Evaluation. Am. J. Prev. Med..

[34] Farley T.A., Rice J., Bodor J.N., Cohen D.A., Bluthenthal R.N., Rose D. (2009). Measuring the Food Environment: Shelf Space of Fruits, Vegetables, and Snack Foods in Stores. J. Urban Health.

[35] Baker J., Parasuraman A., Grewal D., Voss G.B. (2002). The Influence of Multiple Store Environment Cues on Perceived Merchandise Value and Patronage Intentions. J. Mark..

[36] Bava C.M., Jaeger S.R., Dawson J. (2009). In-store influences on consumers’ grocery purchasing decisions: A qualitative investigation. J. Cust. Behav..

[37] Bodor J.N., Rose D., Farley T.A., Swalm C., Scott S.K. (2008). Neighbourhood fruit and vegetable availability and consumption: The role of small food stores in an urban environment. Public Health Nutr..

[38] Thornton L.E., Cameron A.J., McNaughton S.A., Waterlander W.E., Sodergren M., Svastisalee C., Blanchard L., Liese A.D., Battersby S., Carter M.-A. (2013). Does the availability of snack foods in supermarkets vary internationally?. Int. J. Behav. Nutr. Phys. Act..

[39] Miller C., Bodor J., Rose D. (2012). Measuring the food environment: A systematic technique for characterizing food stores using display counts. J. Environ. Public Health.

[40] Heinrich K.M., Li D., Regan G.R., Howard H.H., Ahluwalia J.S., Lee R.E. (2012). Store and restaurant advertising and health of public housing residents. Am. J. Health Behav..

[41] Leeflang P., Parreño-Selva J. (2012). Cross-category demand effects of price promotions. J. Acad. Mark..

[42] Laska M.N., Borradaile K.E., Tester J., Foster G.D., Gittelsohn J. (2009). Healthy food availability in small urban food stores: A comparison of four US cities. Public Health Nutr..

[43] Connell C.L., Yadrick M.K., Simpson P., Gossett J., McGee B.B., Bogle M.L. (2007). Food Supply Adequacy in the Lower Mississippi Delta. J. Nutr. Educ. Behav..

[44] Krukowski R.A., West D.S., Harvey-Berino J., Elaine Prewitt T. (2010). Neighborhood Impact on Healthy Food Availability and Pricing in Food Stores. J. Community Health.

[45] Andreyeva T., Blumenthal D.M., Schwartz M.B., Long M.W., Brownell K.D. (2008). Availability and prices of foods across stores and neighborhoods: The case of New Haven, Connecticut. Health Aff..

[46] Cohen J. (1960). A coefficient of agreement for nominal scales. Educ. Psychol. Meas..

[47] McGraw K., Wong S. (1996). Forming inferences about some intraclass correlation coefficients. Psychol. Methods.

[48] Landis J., Koch G. (1977). The measurement of observer agreement for categorical data. Biometrics.

[49] Gittelsohn J., Song H.-J., Suratkar S., Kumar M.B., Henry E.G., Sharma S., Mattingly M., Anliker J.A. (2010). An Urban Food Store Intervention Positively Affects Food-Related Psychosocial Variables and Food Behaviors. Health Educ. Behav..

[50] Gittelsohn J., Dyckman W., Frick K., Boggs M., Haberle H., Alfred J., Vastine A., Palafox N. (2007). A pilot food store intervention in the Republic of the Marshall Islands. Pac. Health Dialogue.

[51] Lee R.M., Rothstein J.D., Gergen J., Zachary D.A., Smith J.C., Palmer A.M., Gittelsohn J., Surkan P.J. (2015). Process Evaluation of a Comprehensive Supermarket Intervention in a Low-Income Baltimore Community. Health Promot. Pract..

[52] Baquero B., Linnan L., Laraia B.A., Ayala G.X. (2014). Process Evaluation of a Food Marketing and Environmental Change Intervention in Tiendas That Serve Latino Immigrants in North Carolina. Health Promot. Pract..

[53] Ayala G.X., Baquero B., Laraia B.A., Ji M., Linnan L. (2013). Efficacy of a store-based environmental change intervention compared with a delayed treatment control condition on store customers’ intake of fruits and vegetables. Public Health Nutr..

[54] Holmes A.S., Estabrooks P.A., Davis G.C., Serrano E.L. (2012). Effect of a Grocery Store Intervention on Sales of Nutritious Foods to Youth and Their Families. J. Acad. Nutr. Diet..

[55] Thorndike A.N., Bright O.-J.M., Dimond M.A., Fishman R., Levy D.E. (2017). Choice architecture to promote fruit and vegetable purchases by families participating in the Special Supplemental Program for Women, Infants, and Children (WIC): Randomized corner store pilot study. Public Health Nutr..

[56] Cavanaugh E., Green S., Mallya G., Tierney A., Brensinger C., Glanz K. (2014). Changes in food and beverage environments after an urban corner store intervention. Prev. Med..

[57] Houghtaling B., Serrano E.L., Kraak V.I., Harden S.M., Davis G.C., Misyak S.A. (2019). A systematic review of factors that influence food store owner and manager decision making and ability or willingness to use choice architecture and marketing mix strategies to encourage healthy consumer purchases in the United States, 2005–2017. Int. J. Behav. Nutr. Phys. Act..

[58] Ayala G.X., D’Angelo H., Gittelsohn J., Horton L., Ribisl K., Sindberg L.S., Olson C., Kharmats A., Laska M.N. (2017). Who is behind the stocking of energy-dense foods and beverages in small stores? The importance of food and beverage distributors. Public Health Nutr..

[59] Curran S., Gittelsohn J., Anliker J., Ethelbah B., Blake K., Sharma S., Caballero B. (2005). Process evaluation of a store-based environmental obesity intervention on two American Indian Reservations. Health Educ. Res..

[60] Lent M.R., Vander Veur S.S., McCoy T.A., Wojtanowski A.C., Sandoval B., Sherman S., Komaroff E., Foster G.D. (2014). A randomized, controlled study of a healthy corner store initiative on the purchases of urban, low-income youth. Obesity.

[61] Liese A.D., Weis K.E., Pluto D., Smith E., Lawson A. (2007). Food Store Types, Availability, and Cost of Foods in a Rural Environment. J. Am. Diet. Assoc..

[62] Brown C.H., Curran G., Palinkas L.A., Aarons G.A., Wells K.B., Jones L., Collins L.M., Duan N., Mittman B.S., Wallace A. (2017). An Overview of Research and Evaluation Designs for Dissemination and Implementation. Annu. Rev. Public Health.

[63] Hendricks Brown C., Ten Have T.R., Jo B., Dagne G., Wyman P.A., Muthén B., Gibbons R.D. (2009). Adaptive Designs for Randomized Trials in Public Health. Annu. Rev. Public Health.

[64] Almirall D., Nahum-Shani I., Sherwood N.E., Murphy S.A. (2014). Introduction to SMART designs for the development of adaptive interventions: With application to weight loss research. Transl. Behav. Med..

[65] Sigurdsson V., Larsen N.M., Gunnarsson D. (2011). An in-store experimental analysis of consumers’ selection of fruits and vegetables. Serv. Ind. J..

[66] Caspi C.E., Lenk K., Pelletier J.E., Barnes T.L., Harnack L., Erickson D.J., Laska M.N. (2017). Food and beverage purchases in corner stores, gas-marts, pharmacies and dollar stores. Public Health Nutr..

[67] Castro I.A., Majmundar A., Williams C.B., Baquero B. (2018). Customer Purchase Intentions and Choice in Food Retail Environments: A Scoping Review. Int. J. Environ. Res. Public Health.

[68] Mikkelsen B.E., Novotny R., Gittelsohn J. (2016). Multi-level, multi-component approaches to community based interventions for healthy living—A three case comparison. Int. J. Environ. Res. Public Health.

[69] Wallerstein N., Duran B. (2010). Community-based participatory research contributions to intervention research: The intersection of science and practice to improve health equity. Am. J. Public Health.

