# Supplementary material for: Evaluation of Store Environment Changes of an In-Store Intervention to Promote Fruits and Vegetables in Latino/Hispanic-Focused Food Stores
Source: Int J Environ Res Public Health. 2019 Dec 20;17(1):65. doi: 10.3390/ijerph17010065 (PMC6981808; doi:10.3390/ijerph17010065)
Supplement: Supplementary file 1 [file ijerph-17-00065-s001.pdf]

International Journal of
Environmental Research
and Public Health

Supplementary Material 1: ***El Valor de Nuestra Salud*** **(The Value of Our Health) point-of-purchase promotional materials**

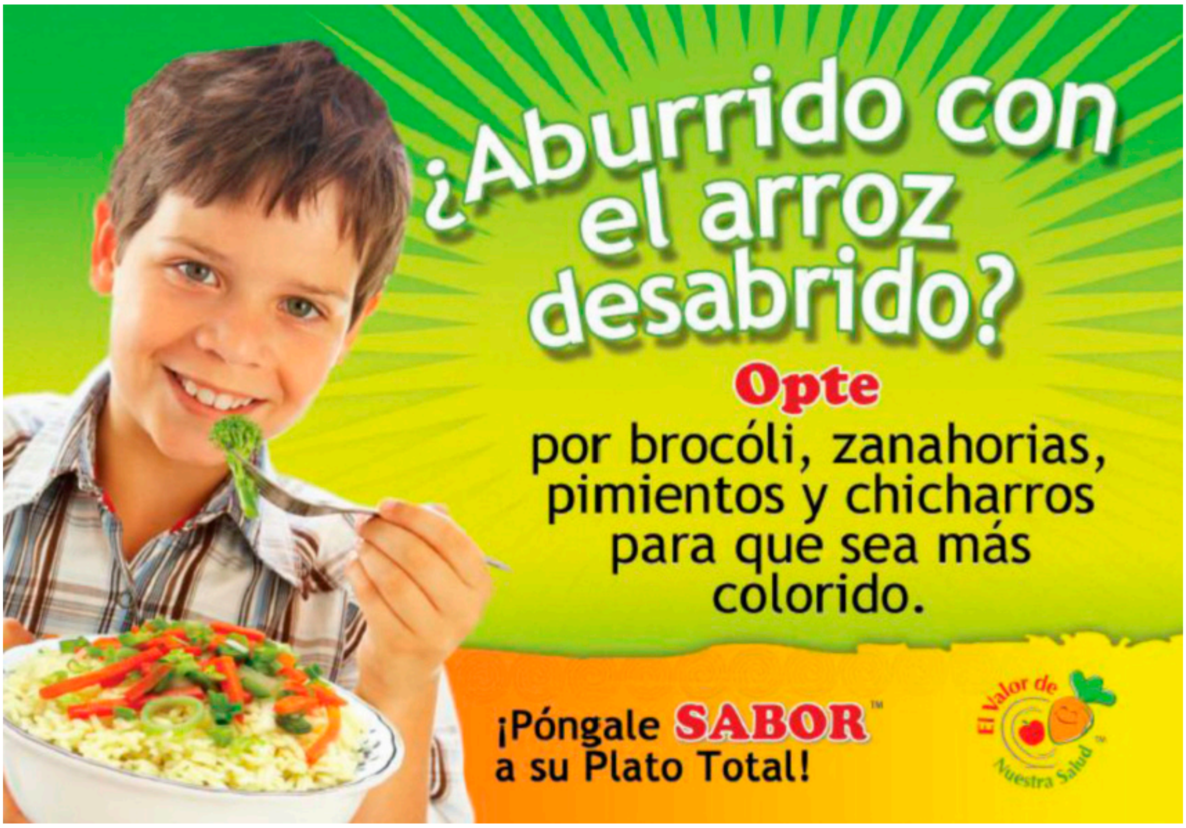

Figure 1S. Shelf dangler

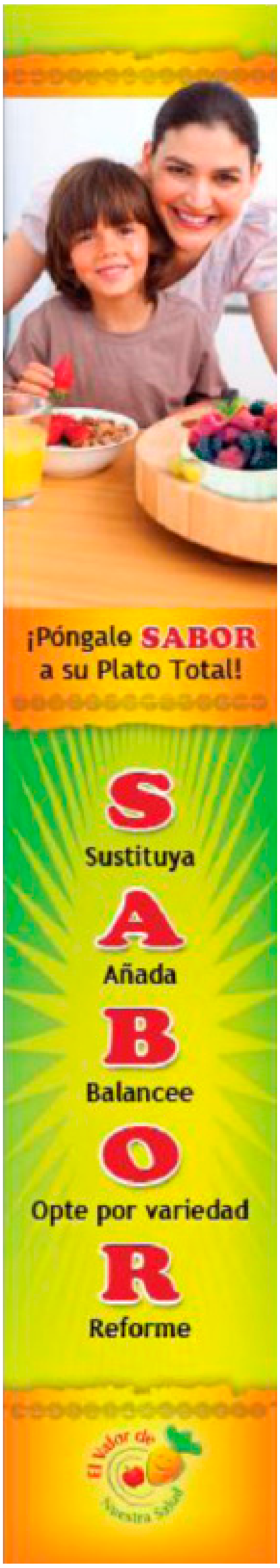

Figure 2S: Aisle violator.
